# Effects of climate variability and urbanization on spatiotemporal patterns of vegetation in the middle and lower Yangtze River Basin, China

**DOI:** 10.3389/fpls.2024.1459058

**Published:** 2024-11-04

**Authors:** Jianxiong Liu, Jing Fu, Jianxin Qin, Baoling Su, Yang Hong

**Affiliations:** ^1^ College of Geography and Tourism, Hengyang Normal University, Hengyang, China; ^2^ Hunan Key Laboratory of Geospatial Big Data Mining and Application, Hunan Normal University, Changsha, China; ^3^ International Centre on Space Technologies for Natural and Cultural Heritage (HIST) under the Auspices of United Nations Educational, Scientific and Cultural Organization (UNESCO), Hengyang Base, Hengyang, China

**Keywords:** vegetation, spatiotemporal patterns, influencing factors, future trend, middle and lower Yangtze River basin

## Abstract

Vegetation serves as a crucial indicator of ecological environment and plays a vital role in preserving ecosystem stability. However, as urbanization escalates rapidly, natural vegetation landscapes are undergoing continuous transformation. Paradoxically, vegetation is pivotal in mitigating the ecological and environmental challenges posed by urban sprawl. The middle and lower Yangtze River Basin (MLYRB) in China, particularly its economically thriving lower reaches, has witnessed a surge in urbanization. Consequently, this study explored the spatiotemporal variations of normalized difference vegetation index (NDVI) in the MLYRB, with an emphasis on elucidating the impact of climate change and urbanization on vegetation dynamics. The results indicate that a significant increasing trend in NDVI across the MLYRB from 2000 to 2020, a pattern that is expected to persist. An improvement in vegetation was observed in 94.12% of the prefecture-level cities in the study area, predominantly in the western and southern regions. Temperature and wind speed stand out as dominant contributors to this improvement. Nevertheless, significant vegetation degradation was detected in some highly urbanized cities in the central and eastern parts of the study area, mainly attributed to the negative effects of escalating urbanization. Interestingly, a positive correlation between NDVI and the urbanization rate was observed, which may be largely related to proactive ecological preservation policies. Additionally, global climatic oscillations were identified as a key force driving periodic NDVI variations. These findings hold significant importance in promoting harmonious urbanization and ecological preservation, thereby providing invaluable insights for future urban ecological planning efforts.

## 1 Introduction

Climate warming stands as one of the pressing environmental concerns globally, posing a substantial challenge to achieving the UN Sustainable Development Goals (SDGs). The greenhouse effect, primarily caused by CO_2_ emissions, is a significant contributor to this warming (IPCC, 2014)., Notably, vegetation performs a critical function in sequestering carbon within the global carbon cycle (Chen et al., 2020; Wu et al., 2023), thereby assisting in climate warming mitigation. Meanwhile, the health and distribution of vegetation are paramount in attaining the SDGs. Hence, monitoring vegetation dynamics aids not just in evaluating ecosystem functions but also furnishes vital insights for formulating and executing effective ecological management tactics (Gao et al., 2022).

Under the combined influences of climate change and human intervention, vegetation cover of the underlying surface has undergone substantial transformation (Huang et al., 2021b; Piao et al., 2019). Specifically, there has been a general upward trend in vegetation cover in the mid-latitudes of the Northern Hemisphere (Myneni et al., 1997; Zheng et al., 2021). Likewise, Africa has witnessed a slight increase in vegetation greenness (Umuhoza et al., 2023). A greening trend is also evident in karst areas globally (Huang et al., 2022). In contrast, North America and Southwest Asia have experienced severe vegetation degradation (Lotsch et al., 2005). In China, while there is an overall increase in vegetation, certain areas such as Northeast China, northern Xinjiang Uygur Autonomous Region, and the Qinghai-Tibet Plateau have seen notable vegetation degradation (Zhou et al., 2020). Alarmingly, there are indications that vegetation browning may become a more prevalent phenomenon in the foreseeable future (Liu et al., 2023b). Evidently, the intricate interactions between climate change and anthropogenic activities are responsible for the conspicuous regional disparities in vegetation dynamics.

Despite the widespread recognition that both climate change and human intervention affects vegetation dynamics, quantifying the relative contributions of these factors to vegetation changes remains a challenge. Currently, scholars primarily utilize statistical methods such as Pearson correlation (Chu et al., 2019), partial correlation (Xie et al., 2022), and multiple linear regression (Long et al., 2023) to establish a direct linear connection between vegetation patterns and climatic conditions. This process aids in determining the specific role played by climatic variables in vegetation dynamics. In terms of assessing the impact of human intervention on vegetation, common quantitative techniques include the use of geographic detector (Zheng et al., 2021) and residual analysis (Lamchin et al., 2020; Liu et al., 2022; Qi et al., 2019). However, given that the multifaceted nature of anthropogenic impacts, these methods often struggle to precisely distinguish the contribution of specific human-induced factors to vegetation changes, which may lead to potential biases in estimating the influence of anthropogenic activities. Additionally, as spatial distance from human activity areas gradually increases, the impact of anthropogenic factors on vegetation tends to progressively weaken until it disappears completely (Bernier et al., 2017; Yi et al., 2021a).

In recent years, climate change has emerged as the primary driving force behind changes in global vegetation greenness (Lamchin et al., 2020; Lawal et al., 2019). To some extent, increasing temperature can enhance vegetation photosynthesis and extend the growing season (Shen et al., 2014). Meanwhile, precipitation serves as an crucial water source for vegetation growth (Wang et al., 2022b). Nevertheless, the impact and magnitude of temperature and precipitation on vegetation vary greatly across diverse eco-geographical regions. For instance, vegetation growth in arid regions is mainly limited by soil moisture (Du et al., 2019b), whereas temperature plays a pivotal role in high-altitude regions (Li et al., 2019). Importantly, the effect of temperature and precipitation on vegetation does not follow a linear enhancement pattern. Extreme climatic conditions, resulting from excessively high temperatures and heavy precipitation, can negatively affect vegetation growth (Zhang, 2020).

The relationship between urbanization and vegetation evolution is intimate. Rapid urban expansion has resulted in a notable surge of impervious surfaces, thereby exerting a conspicuous negative influence on vegetation (Yan et al., 2019). This phenomenon is evident in numerous cities worldwide (De Carvalho and Szlafsztein, 2019; De La Barrera and Henríquez, 2017; Wu et al., 2019). Conversely, changes in vegetation serve as a mirror, reflecting urbanization’s imprint on the terrain and ecological ambiance. To illustrate, the demolition of vegetation has the potential to spark urban environmental challenges (IPCC, 2014), ultimately impinging on the stability and service functions of urban ecosystems (Alpaidze and Salukvadze, 2023). However, cities ironically offer novel habitats for vegetation. Research indicates that urbanization bears an indirect positive effect on vegetation, evident in the amplified growth of vegetation within urban boundaries over time (Zhang et al., 2022a). In addition, this indirect effect is further shaped by a blend of climatic and anthropogenic factors, which, to a certain degree, counteract the negative direct impact of urbanization.

Previous studies on vegetation dynamics in China primarily focused on geographically delimited areas, particularly emphasizing specific regions such as the Yangtze River Basin (Wang et al., 2022a), the Yellow River Basin (Liu et al., 2023a), the Loess Plateau (Li et al., 2021a), and the Tibetan Plateau (Li et al., 2019). Nevertheless, these macroscopic studies often overlook intricate details concerning vegetation changes. The middle and lower Yangtze River Basin (MLYRB), a densely populated and highly urbanized area in China, highlights the importance of understanding the effects of urban sprawl on vegetation. Additionally, quantitative investigations into the impact of urbanization on vegetation remains scarce. Therefore, the primary goals of this study were to: (1) reveal the unique regional and municipal patterns of past and future vegetation variations in the MLYRB; (2) explore the interplay between vegetation and climate variability; and (3) analyze the impact of urbanization on vegetation changes.

## 2 Materials

### 2.1 Study area

**Figure 1:**
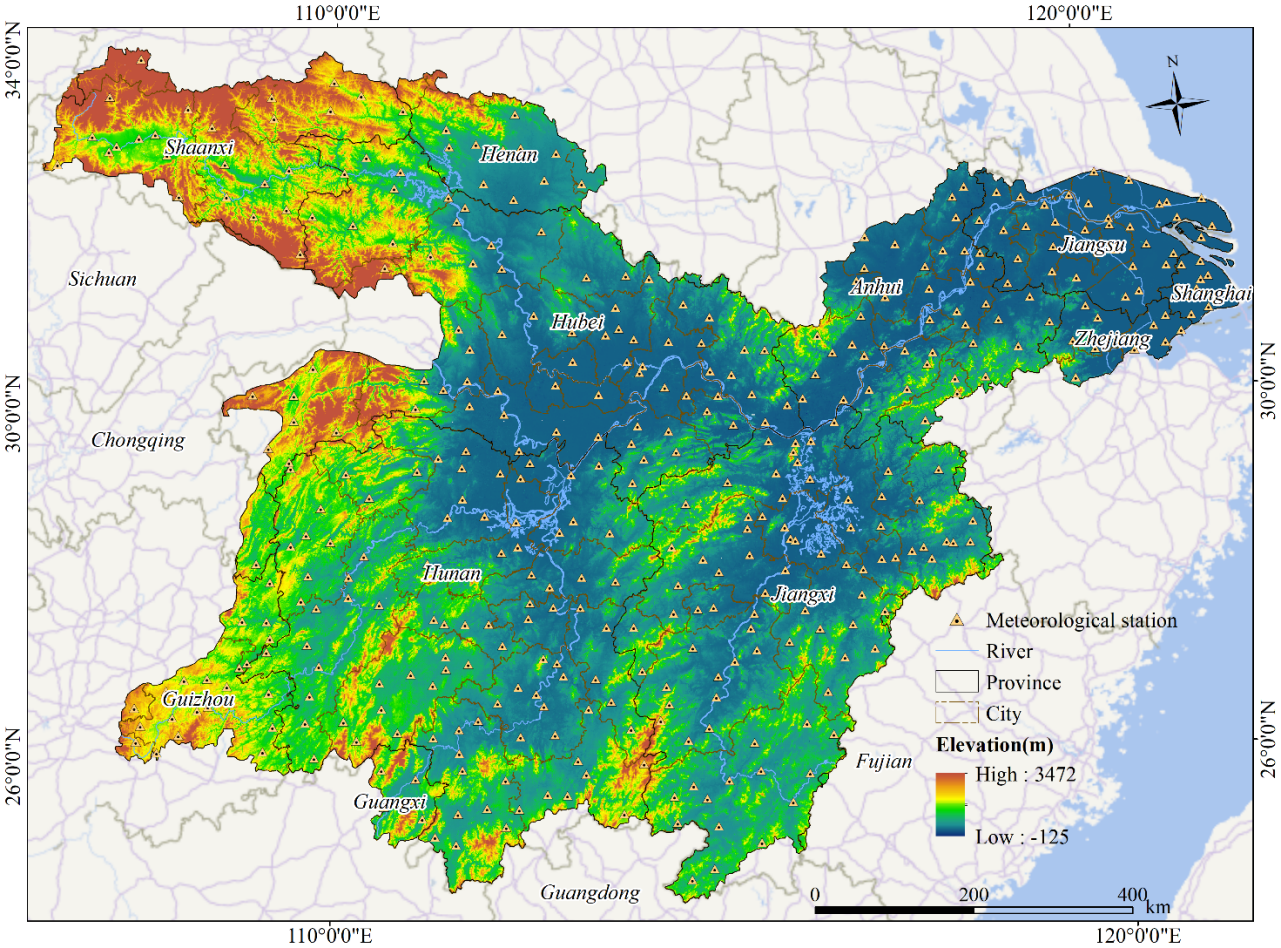
Spatial distribution of provinces, cities, meteorological stations, main rivers, and digital elevation model in the middle and lower Yangtze River Basin, China.

The MLYRB, situated between 24°30′−34°10′ N and 105°52′−122°25′ E, lies in the southern parts of the central and eastern China. This vast region encompasses 13 provincial-level and 68 city-level administrative regions (**Figure 1**), with a total area of approximately 78.64×10^4^ km^2^. By 2020, the urbanization rate in the basin had surpassed 58%, making it as one of China’s most urbanized levels and densely populated areas. The terrain of the MLYRB is predominantly flat, featuring plains such as the Jianghan-Dongting Lake Plain (also known as the Lianghu Plain), Poyang Lake Plain, and Central Anhui Riverside Plain. The MLYRB boasts a dense network of rivers and abundant water resources, including freshwater lakes like Dongting Lake, Poyang Lake, Taihu Lake, and Chaohu Lake. Characterized by a subtropical monsoon climate, the basin experiences moderate temperature and abundant rainfall. The annual average temperature ranges from 8.79 to 20.50 °C, while the yearly precipitation varies between 643.19 mm and 2358.40 mm. These climatic conditions foster healthy vegetation growth, favoring both forests and croplands. However, from August to October every year, as the East Asian summer monsoon shifts southward, abnormal monsoon rain belts can cause floods in MLYRB (Li et al., 2022). Correspondingly, excessive precipitation in the study area can trigger hypoxia in the root systems of surface vegetation, hindering their growth or even causing plant death. Additionally, excessively high summer temperature can elevate vegetation evaporation, potentially leading to droughts that inhibit vegetation growth in the study area (Huang et al., 2021a).

### 2.2 Data

#### 2.2.1 MODIS data

The MODIS normalized difference vegetation index (NDVI) time series data utilized in this study, spanning from 2000 to 2020, were sourced from the Land, Atmosphere Near-real-time Capability for EOS (LANCE) system, accessible at https://ladsweb.modaps.eosdis.nasa.gov/. These data were archived in the HDF format, featuring a temporal resolution of 16 days and a spatial resolution of 250 meters. The MODIS Reprojection Tool, provided by the United States Geological Survey (USGS), facilitated the mosaicking, format conversion, and projection of the MODIS data. To account for missing data in January and February of 2000, multi-year synchronous average values were utilized. Monthly NDVI datasets were created using the maximum-value composites procedure to minimize the effects of cloud cover, atmospheric conditions, and solar zenith angle variability. Subsequently, the mean method was applied to generate annual NDVI datasets, aiming to mitigate the impact of climatic anomalies on NDVI in certain months as much as possible.

Generally, the calculation of NDVI is highly correlated with near-infrared and red bands. Falling within the range of [−1, 1], this index effectively indicates vegetation growth status. Specifically, a negative NDVI value signifies the presence of ground cover, such as clouds, water, or snow. Conversely, a positive value denotes vegetation cover, with values closer to 1 exhibiting a greater degree of coverage. Furthermore, an NDVI nearing 0 suggests the existence of rocks, bare soil, or other unvegetated surfaces.

#### 2.2.2 Climate data

The meteorological data utilized in this study were obtained from the Hunan Meteorological Bureau. It encompassed various climatic variables for the MLYRB during 2000−2020, including the annual average temperature (°C), annual average relative humidity (%), annual average wind speed (m·s^−1^), annual total precipitation (mm), and annual total sunshine duration (h). This dataset was derived from the climate records of 410 Chinese national meteorological stations within the study area. Also, this dataset was interpolated by the Inverse Distance Weighting (IDW) method to generate meteorological raster data with the same projection and resolution as the NDVI dataset, enabling an analysis of the contribution of climatic variables to NDVI changes.

Additionally, this study delved into the influence of global-scale climate oscillations on the periodic variations in NDVI. The specific climate indices examined include the Atlantic Multidecadal Oscillation (AMO), North Atlantic Oscillation (NAO), Pacific Decadal Oscillation (PDO), and Southern Oscillation Index (SOI). These data were sourced from the Physical Science Laboratory of National Oceanic and Atmospheric Administration (NOAA) (https://psl.noaa.gov/) and National Climate Prediction Center of NOAA (https://www.cpc.ncep.noaa.gov/).

#### 2.2.3 Other data

The Digital Elevation Model (DEM) was accessed from the Geospatial Data Cloud Platform (http://www.gscloud.cn/). Administrative division vector data was procured from the Data Center for Resource and Environment Sciences, Chinese Academy of Sciences (http://www.resdc.cn/). Details of El Niño and La Niña events occurring since 2000 were gathered from the National Climate Center of China website (http://cmdp.ncc-cma.net/). In addition, the annual urbanization rate data for each county from 2003 to 2020 was extracted from the *China Statistical Yearbook*, published by the China National Bureau Statistics (http://www.stats.gov.cn/).

## 3 Methods

### 3.1 Trend detection and analysis

#### 3.1.1 Sen’s slope and Mann-Kendall test

The integration of Sen’s slope estimator (SSE) and Mann-Kendall (M-K) significance test has proven highly effective in discerning trends in vegetation cover, be it increasing or decreasing. This combined approach has gained widespread application in hydrological and meteorological research (Tong et al., 2018; Zhang and Jin, 2021). SSE, recognized as a robust non-parametric statistical method (Sen, 1968; Theil, 1992), excels at minimizing the influence of error and outliers in the raw data on statistical outcomes. In this research, this method was applied to estimate the trend in NDVI. A positive or negative value of the estimated result indicates corresponding upward or downward trend in NDVI, respectively. Additionally, the M-K test (Kendall, 1975; Mann, 1945) was leveraged to determine the statistical significance of these NDVI trends. In the context of a two-tailed trend test, if the standardized test statistic meets or exceeds the thresholds of 1.96 and 2.58, it signifies that the observed trend has passed the significance test, corresponding to confidence levels of α=0.05 and α=0.01 , respectively.

#### 3.1.2 Seasonal Autoregressive Integrated Moving Average Model

The Autoregressive Integrated Moving Average (ARIMA) model, originally introduced by Box and Jenkins (Box and Jenkins, 1970), stands as a prevalent time series forecasting model. Given that time series data often exhibits patterns of periodicity or seasonality, the ARIMA model can be extended to the Seasonal Autoregressive Integrated Moving Average (SARIMA) model. This method is denoted as $SARIMA(p,d,q){\left(P,D,Q\right)}_{S}$ , wherein *p* and *P* represent the autoregressive order and the seasonal autoregressive order, respectively. Similarly, *d* and *D* indicate the trend differences and the seasonal difference order, while *q* and *Q* signify the moving average order and the seasonal moving average order, respectively. Additionally, *S* depicts the seasonal cycle length. Regarding the NDVI time series training set Xt employed in this study, the SARIMA model can be expressed as follows:


(1) $$\nabla^{d}\nabla_{S}^{D}X_{t}=\frac{\theta (B)\Theta_{S}(B)}{\phi (B)\Phi_{S}(B)}a_{t}$$

where $\nabla^{d}$ refers to the *d*-order difference; $\nabla_{S}^{D}$ is the *D*-order seasonal difference with *S* as the period; $\theta (B)=1-\theta_{1}B-\ldots -\theta_{q}B^{q}$ denotes the *q*-order moving average coefficient polynomial, *B* represents the lag operator, and $B^{k}X_{t}=X_{t}-k$ ; $\phi (B)=1-\phi_{1}B-\ldots -\phi_{p}B^{p}$ signifies the *p*-order autoregressive coefficient polynomial; $\Theta S(B)=1-\Theta_{1}B^{S}-\ldots -\Theta_{Q}B^{QS}$ corresponds to the *Q*-order seasonal moving average coefficient polynomial; $\Phi S(B)=1-\Phi_{1}B^{S}-\ldots -\Phi_{P}B^{PS}$ depicts the *P*-order seasonal autoregressive coefficient polynomial; and at indicates the random disturbance of error term at time *t*.

### 3.2 Attribution analysis

#### 3.2.1 Multiple linear regression

Compared to the single-factor correlation, multi-factor analysis offers a more comprehensive understanding of the relationship between NDVI and diverse climatic factors. The multiple linear regression (MLR) model is instrumental in examining the interplay between a dependent variable and multiple independent variables (Fu et al., 2022, 2024). In this study, the model was employed to evaluate how temperature, precipitation, relative humidity, sunshine duration, and wind speed affect NDVI. To neutralize the impacts caused by the different dimensions of climatic variables, it is necessary to standardize these variables before applying the MLR. The formula for MLR can be described below:


(2) $$Y_{\text{NDVI}}=a_{0}+a_{1}X_{\text{Tem}}+a_{2}X_{\text{Pr}}+a_{3}X_{\text{RH}}+a_{4}X_{\text{SD}}+a_{5}X_{\text{WS}}$$

where $Y_{\text{NDVI}}$ refers to the NDVI time series; $X_{\text{Tem}}$ , $X_{\text{Pr}}$ , $X_{\text{RH}}$ , $X_{\text{SD}}$ , and $X_{\text{WS}}$ represent the time series for temperature, precipitation, relative humidity, sunshine duration, and wind speed, respectively; $a_{0}$ denotes a constant; $a_{i}\;(i=1,2,\ldots ,5)$ is the standard regression coefficients. The absolute values of these coefficients indicate the significance of each climatic variable to NDVI. A larger absolute value of the coefficient implies a greater impact of that particular climatic variable. The relative contribution of various climatic variables to NDVI can be determined by computing the ratio of the absolute value of each variable’s coefficient to the sum of the absolute values of all climatic variable coefficients.

#### 3.2.2 Cross wavelet transform and wavelet coherence

Cross wavelet transform (XWT) integrates wavelet transform (WT) with cross-spectrum analysis, enabling the precise identification of the significant interactions between two time series across various time-frequency domains. Furthermore, XWT reveals the correlation consistency among sequences and elucidates their phase relationships in the time-frequency space. Assuming $W_{n}^{x}(s)$ and $W_{n}^{y}(s)$ represent the XWT of two time series, *X* and *Y* respectively, and their cross wavelet power spectrum (CWPS) was defined as (Torrence and Compo, 1998)


(3) $$W_{n}^{xy}(s)=W_{n}^{x}(s)W_{n}^{y*}(s)$$

where the absolute value of the left-hand side of the equation corresponds to the density of the CWPS. A larger absolute value indicates a more significant correlation between the high-energy regions of two time series.

Provided that the expected spectra of both time series *X* and *Y* are red noise spectra, denoted as $P_{k}^{x}$ and $P_{k}^{y}$ respectively, the distribution relationship of the CWPS can be expressed as follows:


(4) $$\left|\frac{W_{n}^{x}(s)W_{n}^{y*}(s)}{\sigma_{x}\sigma_{y}}\right|=\frac{Z_{v}(p)}{v}\sqrt{P_{k}^{x}P_{k}^{y}}$$

where $\sigma_{x}$ , $\sigma_{y}$ represent the standard deviations of time series *X* and *Y*, respectively; *v* denotes the degrees of freedom for the Morlet wavelet transform, specifically set at 2. If the left-hand side of the equation exceeds the upper confidence limit of the 95% red noise power spectrum, it is considered to have passed the significance level test at a=0.05 . Furthermore, $Z_{v}(p)$ refers to the confidence level linked to the probability *p*, and $Z_{2}(95\%)=3.999$ at the significance level of a=0.05 .

Wavelet coherence (WTC) addresses the limitations of cross wavelet in detecting correlations between time series, particularly in low-energy region. The WTC can be written as


(5) $$R_{n}^{2}(s)=\frac{{\left|S\left(s^{-1}W_{n}^{xy}\left(s\right)\right)\right|}^{2}}{S\left(s^{-1}{\left|W_{n}^{x}\left(s\right)\right|}^{2}\right)\cdot S\left(s^{-1}{\left|W_{n}^{y}\left(s\right)\right|}^{2}\right)}$$

where *S* refers to the smoother; *s* signifies the companion scaling of wavelet function; || represents modulus for a complex number; ${\left|S\left(s^{-1}W_{n}^{xy}\left(s\right)\right)\right|}^{2}$ denotes the cross product of wave amplitude at certain frequency from two time series; $S\left(s^{-1}{\left|W_{n}^{x}\left(s\right)\right|}^{2}\right)$ and $S\left(s^{-1}{\left|W_{n}^{y}\left(s\right)\right|}^{2}\right)$ are the amplitudes of vibration waves in two time series.

#### 3.2.3 Pearson correlation

Pearson correlation analysis is a wide-used statistical method. The Pearson correlation coefficient can effectively express the degree and direction of a relationship, thereby reflecting connections between different elements (Pearson, 1920). In this research, this method was utilized to analyze the correlation between the urbanization rate and NDVI from 2003 to 2020. The formula for the calculation is as follows:


(6) $$R_{xy}=\frac{\sum_{i=1}^{n}\left[\left(x_{i}-\bar{x}\right)\left(y_{i}-\bar{y}\right)\right]}{\sqrt{\sum_{i=1}^{n}{\left[\left(x_{i}-\bar{x}\right)\left(y_{i}-\bar{y}\right)\right]}^{2}}}$$

where $R_{xy}$ represents the correlation coefficient; $x_{i}$ , $y_{i}$ signify the annual urbanization rate and NDVI, respectively, while $\bar{x}$ , $\bar{y}$ correspond to the average urbanization rate and average NDVI over the study period; *i* refers to the year index (ranging from 1 to 18); *n* indicates the duration of the research, and *n* =18. Generally, if $R_{xy}$ > 0, it suggests a positive correlation between the two variables; conversely, a negative coefficient describes a negative correlation. Also, the closer the absolute value of $R_{xy}$ approaches 1, the stronger the positive (or negative) correlation. Additionally, when the correlation coefficient meets the significance test criteria with a significance level of 95% or above, it demonstrates a statistically significant correlation between NDVI and the urbanization rate.

## 4 Results

### 4.1 Temporal variability of NDVI

#### 4.1.1 NDVI variations at the whole-domain scale

**Figure 2:**
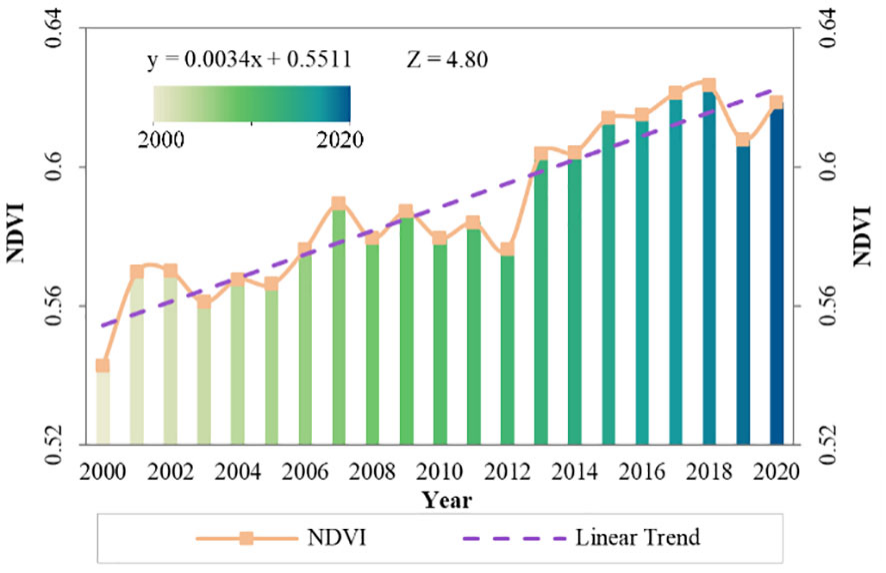
Interannual variations and linear trend of NDVI in the study area, 2000−2020.

The annual average NDVI in the MLYRB rose from 0.54 to 0.62, stabilizing at approximately 0.59. This upward trend was highly significant, with an increment of 0.0034 year^−1^ (*P* < 0.01) (**Figure 2**). The NDVI peaked in 2018. By referencing the *2018 China Ecological Meteorological Bulletin* and the meteorological data of this study, it becomes evident that the majority of the study area enjoyed ample water and warmth in 2018, while meteorological disasters such as droughts and heavy rains had minimal impact. Such climatic conditions are conducive to vegetation growth, and China’s ecological projects have borne positive outcomes. This could explain why the NDVI attained the highest value in 2018. Conversely, the lowest NDVI was occurred in 2000. This can be attributed to the heavy emphasis on economic development during that period, coupled with the lack of comprehensive environmental safeguards and the overexploitation of vegetation resources. The most rapid growing phase of NDVI occurred between 2012 and 2018 (0.0067 year^−1^), followed by the period during 2000−2007 (0.0042 year^−1^). In contrast, the steepest decline in NDVI was witnessed from 2018 to 2020 (−0.0025 year^−1^), preceded by a milder drop from 2007 to 2012 (−0.0017 year^−1^).

#### 4.1.2 NDVI variations at the city scale

**Figure 3:**
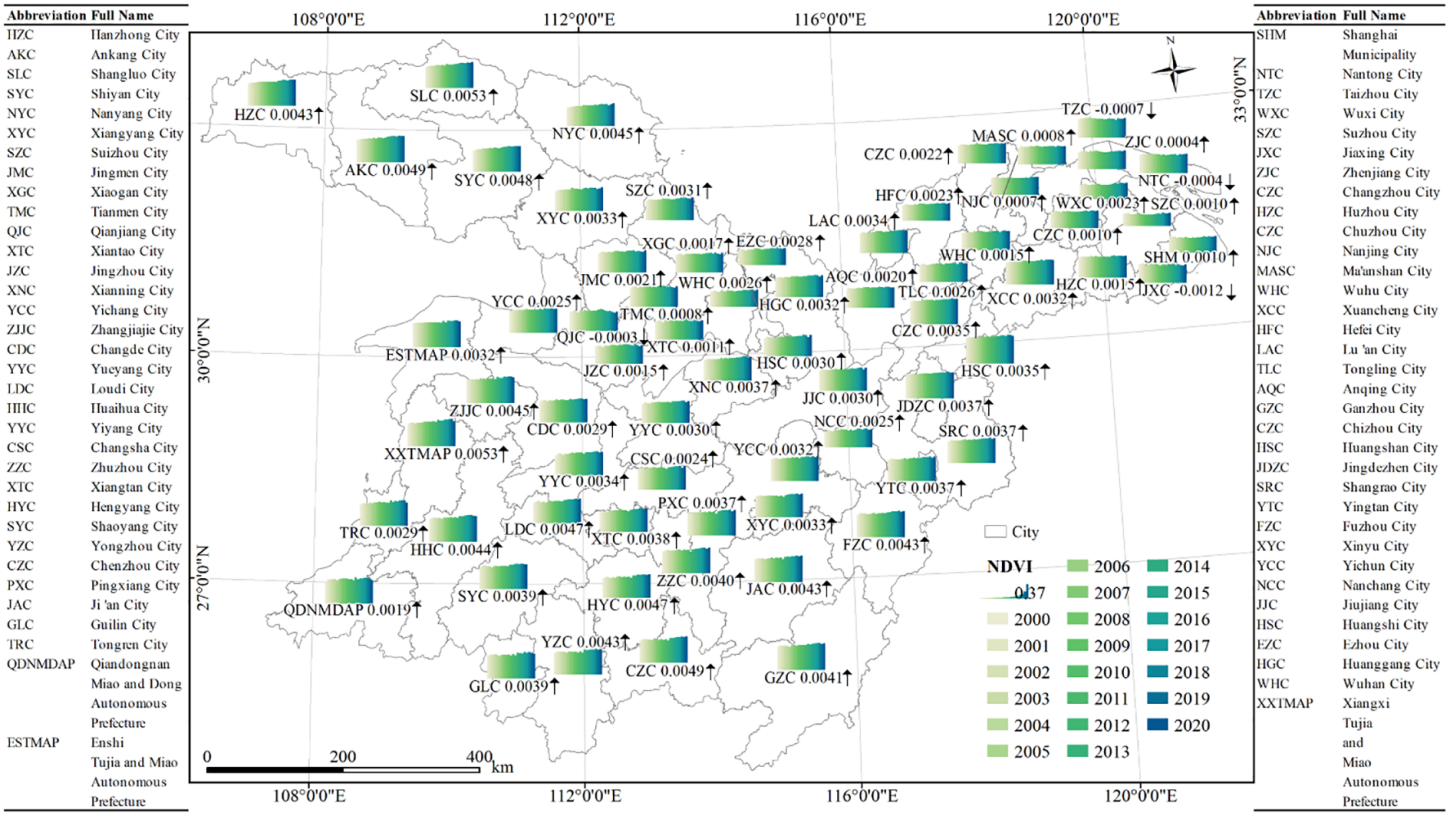
Temporal variations of NDVI at the city scale in the study area, 2000−2020. The arrow symbols, specifically ↑ and ↓, denote upward and downward trends, respectively. HZC stands for Hangzhou City, with similar abbreviations employed for the other cities represented.

As shown in **Figure 3**, five cities in the MLYRB with the highest NDVI over the past 21 years were Huangshan, Jingdezhen, Ganzhou, Enshi Tujia and Miao Autonomous Prefecture (ETMAP), and Huaihua in China. These cities boasted NDVI values of 0.705, 0.664, 0.662, 0.658, 0.655, respectively. In contrast, the lowest NDVI values were observed in Suzhou and Wuxi, at 0.311 and 0.392, respectively. Notably, the NDVI in the central-western region of the study area is significantly higher than that in the eastern region.

Regarding the growth rate of NDVI, most cities displayed an upward trend, accounting for 94.12% of the total cities in the study area. Specifically, Shangluo, Xiangxi Tujia and Miao Autonomous Prefecture, Chenzhou, Ankang, and Shiyan exhibited pronounced growth trends, with rates of 0.0053 year^−1^, 0.0053 year^−1^, 0.0049 year^−1^, 0.0049 year^−1^, and 0.0048 year^−1^, respectively. The Yangtze River Delta region (YRD) stands out for its high urbanization level and consequent vegetation degradation. Within this region, Jiaxing experienced the steepest NDVI decline at −0.0012 year^−1^, followed by Taizhou (−0.0007 year^−1^) and Nantong (−0.0004 year^−1^). In addition, Qianjiang also manifested a downward trend in NDVI with a decrease rate of −0.0003 year^−1^.

### 4.2 Spatial patterns of NDVI

**Figure 4:**
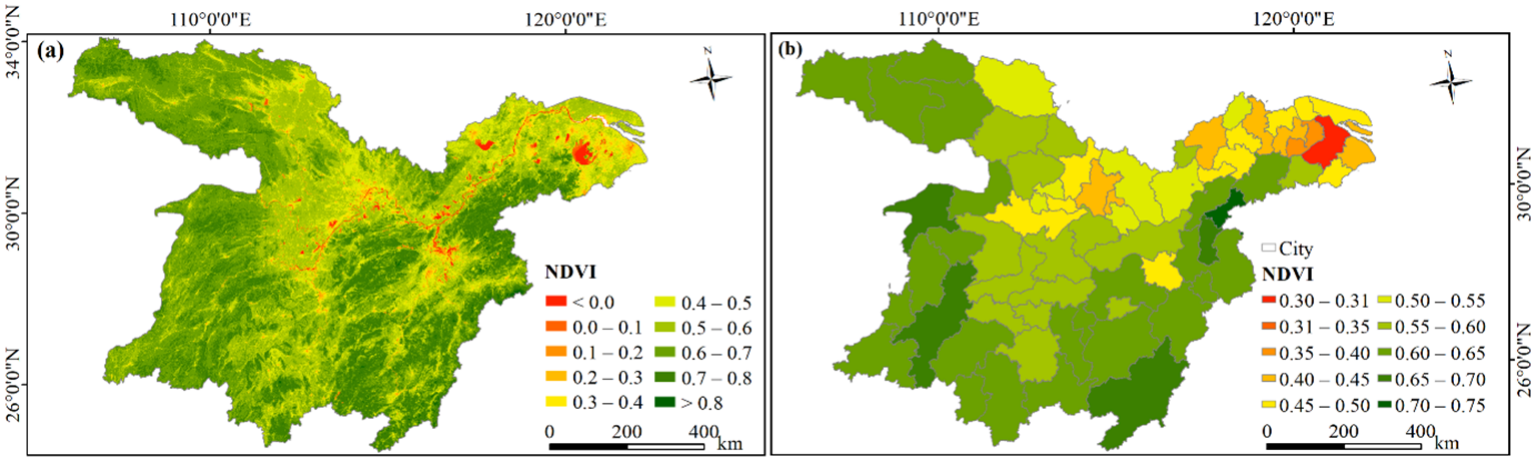
**(A)** Spatial pattern of the average annual NDVI in the study area, 2000−2020. **(B)** Pixel-based statistics of average NDVI at the city scale.

Overall, the majority of the MLYRB was well-covered with vegetation (**Figure 4A**). Spatially, NDVI predominantly adhered to a “high in the west and low in the east, high in the south and low in the north” pattern, primarily attributed to the advanced urbanization along the Yangtze River and in the lower reaches of the Yangtze River Basin (Zheng et al., 2021). The areas with high NDVI values (> 0.6) accounted for 51.83% of the study area (**Figure 4A**). Precisely 22 cities exhibited an NDVI between 0.60 and 0.65, mainly located in the western part of the study area and central-southern part of eastern region (**Figure 4B**). Among these, Huangshan boasted the highest NDVI, followed by Jingdezhen, then Ganzhou, the ETMAP, and Huaihua. The areas with low NDVI values (< 0.2) comprised 1.87% of the study area (**Figure 4A**). On the city scale, NDVI was relatively lower in Wuhan urban circle and the YRD (**Figure 4B**).

### 4.3 Spatial variability of NDVI

#### 4.3.1 Trajectory of NDVI centroid migration

**Figure 5:**
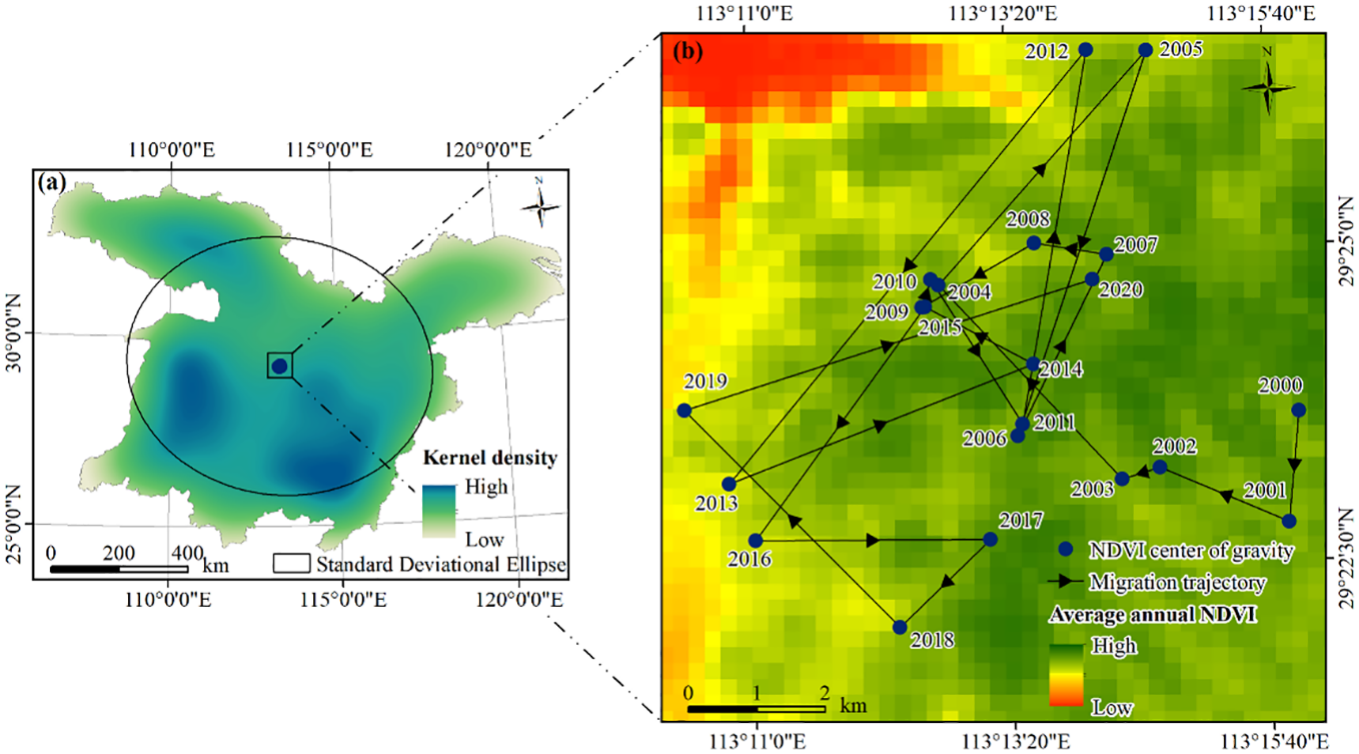
**(A)** Kernel density and standard deviational ellipse of annual NDVI. **(B)** Spatial variations in NDVI centroid migration within the study area, 2000−2020.

As shown in **Figure 5A**, the NDVI kernel density gradually decreased from the southwest to the northeast. Regions with high kernel density were predominantly situated in the central and southern parts of the MLYRB. Characterized by robust vegetation growth and dense vegetation cover, these regions were primarily composed of forests and croplands. Between 2000 and 2020, the geographic center of NDVI in the MLYRB continuously migrated (**Figure 5B**). The longitude of the centroid ranged between 113°10′23.78′′ E and 113°15′56.20′′ E, and the latitude extended from 29°22′1.01′′ N to 29°26′32.91′′ N. Overall, the NDVI centroid coordinates moved from 113°15′56.20′′ E, 29°23′40.29′′ N in 2000 to 113°14′5.85′′ E, 29°24′44.14′′ N in 2020, following a fluctuating trajectory. This migratory trend indicates significant interannual variability in NDVI in the study area. Notably, the migrations during 2005−2006, 2011−2012, 2012−2013, and 2019−2020 were particularly pronounced, covering distances of approximately 5.92 km, 5.54 km, 8.20 km, and 6.25 km, respectively. These distances exhibit an expansion in vegetation cover across a wide area of the study area during these specific periods.

#### 4.3.2 Spatial variation of NDVI

**Figure 6:**
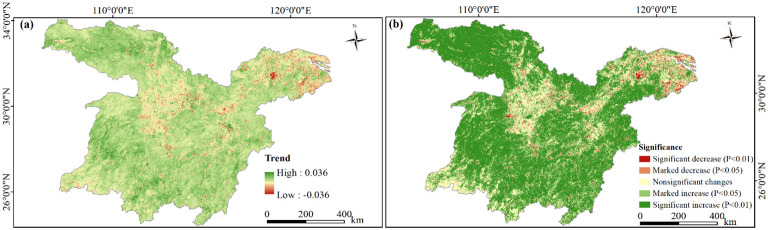
Variability and trends of NDVI in the study area, 2000−2020. **(A)** Spatial patterns of pixel-by-pixel NDVI variations; **(B)** significance of NDVI trends.

As shown in **Figure 6A**, the change rate of NDVI in the MLYRB during 2000−2020 ranged from −0.0362 to 0.0365 per year. The majority of the study area, specifically 89.28%, demonstrated an upward trend in NDVI. These areas were mainly observed in the Qinling-Daba mountainous area and its adjacent areas (southern Shaanxi, southern Henan, and northwestern Hubei), as well as the Wuling-Xuefeng Mountain ranges (western Hunan), and Jiangnan Hills. Conversely, the areas where NDVI showed a downward trend accounted for 10.72%, primarily concentrated in central Hubei, northern Hunan, southern Anhui, and highly urbanized regions in Jiangsu, Zhejiang, and Shanghai Municipality.

According to **Figure 6B**, the majority of the study area (62.65%) exhibited a significant increase in NDVI, followed by regions showing nonsignificant changes (23.81%) and marked increases (9.97%). Conversely, areas with significant decreases and marked decreases only accounted for 3.57%. Specifically, the Dongting Lake Basin (DLB), Poyang Lake Basin (PLB), and Han River Basin (HRB) predominantly featured a significant upward NDVI trend. Regions with marked NDVI increases were dispersed across the study area. Significant NDVI decreases were primarily identified in Lixian and Linli Counties of Changde, Dantu County of Ma’anshan, Xuanzhou District of Xuancheng, Gaochun District of Nanjing, and the vicinity of Taihu Lake. Notably, the Wuhan Metropolitan Area (WMA), Changsha-Zhuzhou-Xiangtan urban agglomeration (CZTUA), Poyang Lake urban agglomeration (PLUA), and the YRD predominantly exhibited a marked decreasing NDVI trend.

### 4.4 Attribution analysis of NDVI variability

#### 4.4.1 Impact of climate change on NDVI

**Figure 7:**
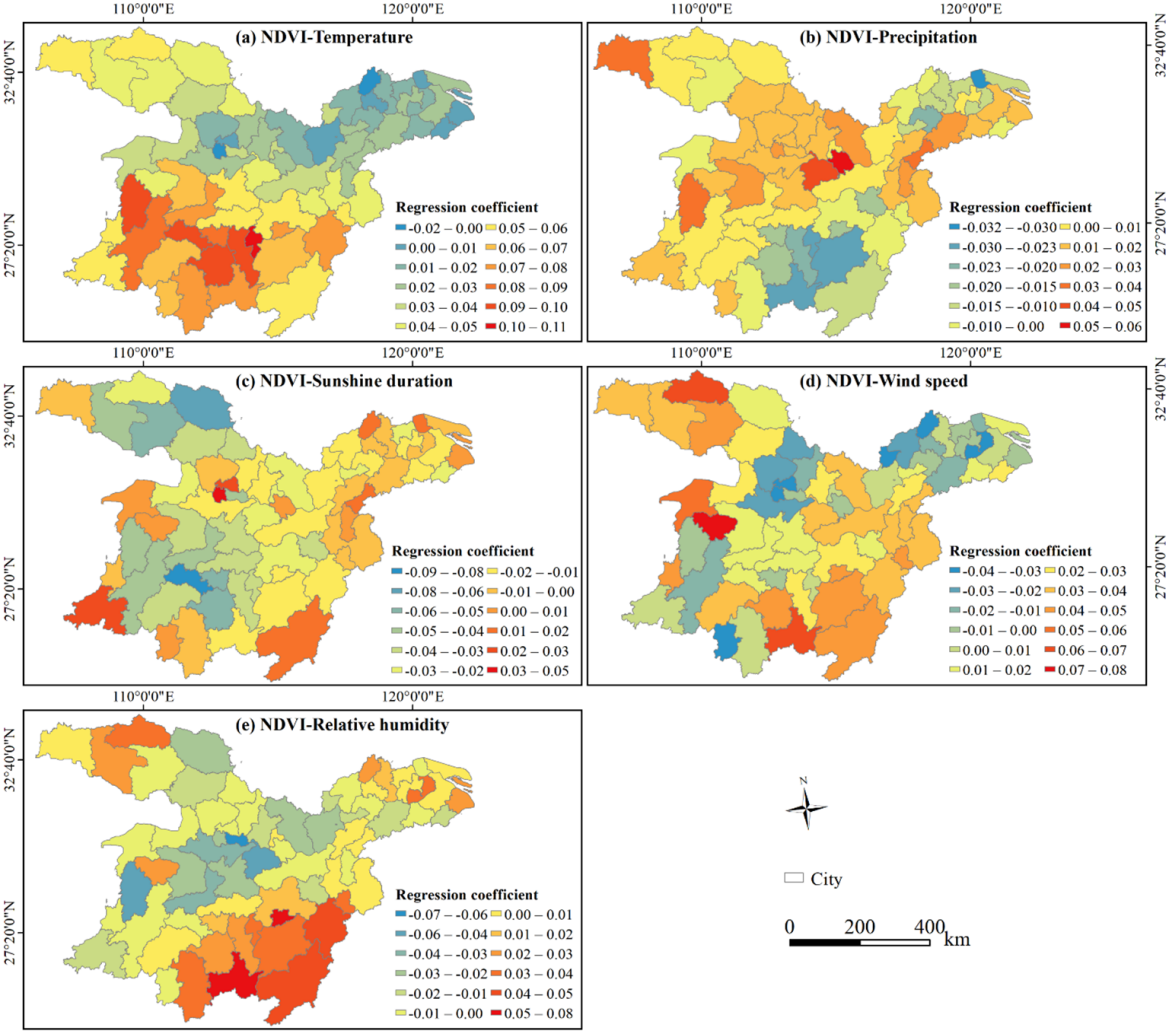
Spatial distribution of regression coefficients between NDVI and climatic variables at the city scale in the study area, 2000−2020. **(A)** Correlation between NDVI and Temperature; **(B)** correlation between NDVI and precipitation; **(C)** correlation between NDVI and sunshine duration; **(D)** correlation between NDVI and wind speed; and **(E)** correlation between NDVI and relative humidity.

The regression coefficients indicate a correlation between NDVI and climatic variables, highlighting notable spatial differences among cities. For example, excluding Qianjiang and Chuzhou, the NDVI of the other 66 cities showed a positive correlation with temperature (**Figure 7A**). Similarly, the NDVI of 62% of the cities positively correlated with precipitation, with Huangshi exhibiting the strongest positive correlation (**Figure 7B**). Notably, the NDVI of 81% of the cities demonstrated a negative correlation with sunshine duration, Loudi exhibiting the strongest negative one (**Figure 7C**). Evidently, the correlation between NDVI and wind speed was negative in cities situated along the southeast-northwest axis, while being positive on both sides of the axis (**Figure 7D**). Regarding relative humidity (**Figure 7E**), 54% of the cities indicated a negative correlation with NDVI, with Xiantao presenting the strongest correlation; and the other cities exhibited a positive correlation, with Xinyu and Chenzhou showing strong correlations.

**Figure 8:**
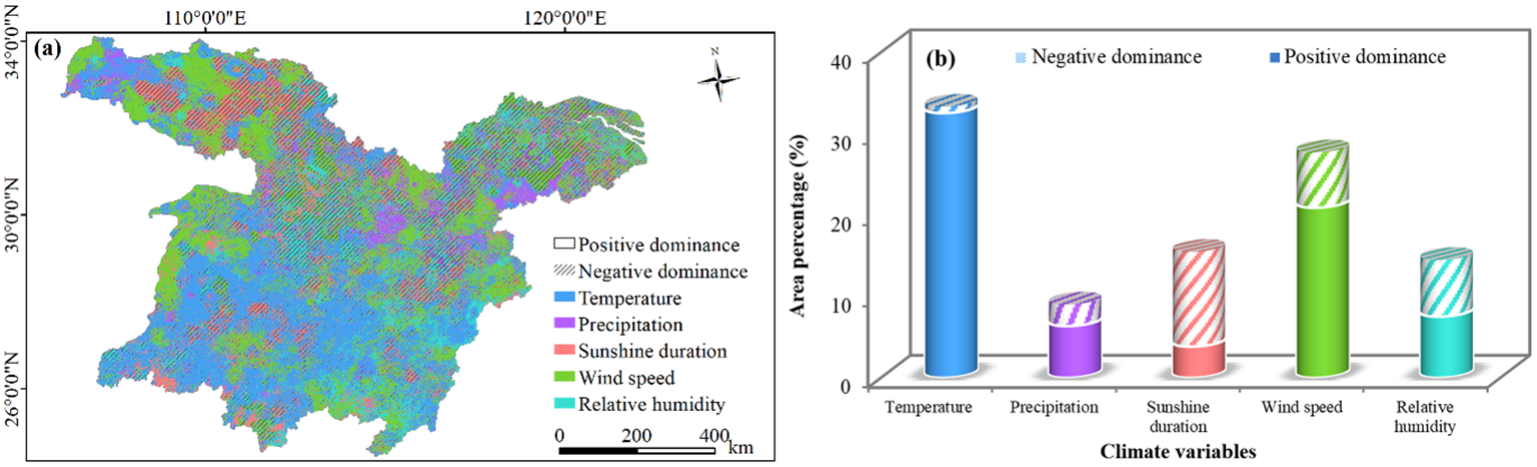
**(A)** Spatial pattern of the dominant factors influencing vegetation changes in the study area, 2000−2020. **(B)** Proportions of positive and negative dominant areas for climatic variables based on pixel statistics.

As shown in **Figure 8**, the areas where NDVI demonstrated a positive correlation with climatic variables accounted for 71.20% of the study area. Aside from sunshine duration, the correlations between other variables (i.e., temperature, wind speed, sunshine duration, relative humidity, and precipitation) and NDVI were mainly positive. Specifically, temperature exerted the most significant influence on vegetation changes, affecting 33.33% of the study area. The regions where NDVI positively correlated with temperature were mainly distributed in the DLB, PLB, Hanzhong, certain parts of Shangluo, Suizhou, Xiaogan, and northern Huanggang. Wind speed ranked as the second dominated factor of NDVI, encompassing 27.71%. The positive correlation areas of NDVI and temperature were primarily found in Shanghai, the ETMAP, parts of Shiyan, central Huanggang, Chizhou, and Shaoyang, western Xiangyang and Huangshan, southern Chenzhou, and eastern Shangrao.

Additionally, sunshine duration, relative humidity, and precipitation also exerted notable effects on NDVI, accounting for 15.47%, 14.48% and 9.01% of the study area, respectively. NDVI negatively correlated with Sunshine duration in specific areas like central Huaihua and Xinhua County of Loudi, Shiyan, central Ankang, Nanyang, and the intersection of Yichun, and Nanchang. The areas where NDVI positively correlated with relative humidity were mainly observed in Quanjiao and Dingyuan Counties of Chuzhou, Shushan District of Hefei, Jing County of Xuancheng, Lishui District of Nanjing, Pucheng County of Nanping, Zixi County of Fuzhou, Guidong County and Zixing City of Chenzhou, Yongding District of Zhangjiajie, Jingshan of Jingmen, Liuba County of Hanzhong, Zhashui County of Shangluo. The positively correlated areas of NDVI and precipitation were mainly located in southern Xi’an, western and central Hanzhong, eastern Xianning and Huangshi, and southern Xuancheng.

#### 4.4.2 Impact of global-scale climate oscillations on NDVI

**Figure 9:**
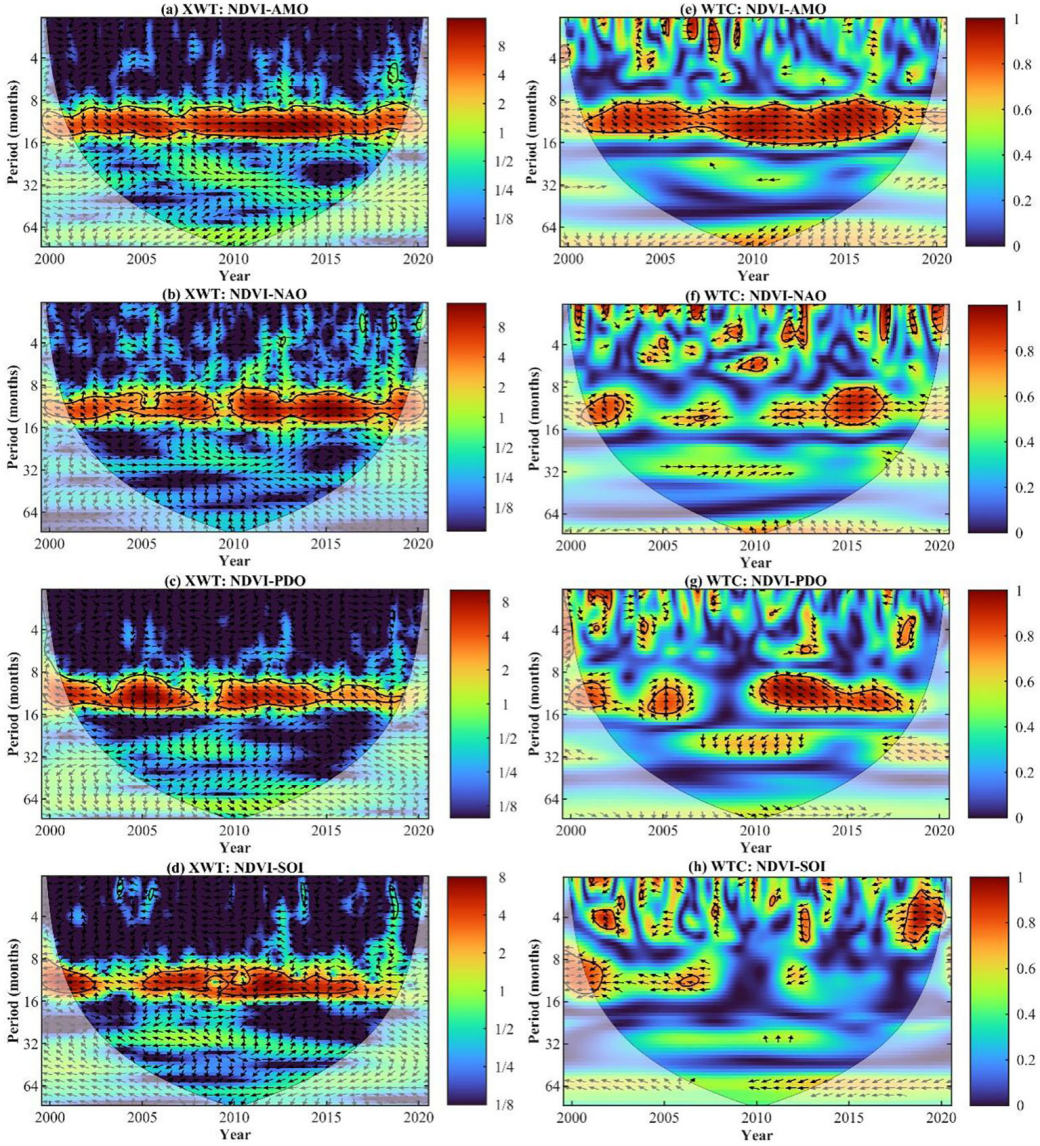
**(A−D)** Cross wavelet power spectrum and **(E−H)** wavelet coherence between NDVI and climate indices (i.e., AMO, Atlantic Multidecadal Oscillation; NAO, North Atlantic Oscillation; PDO, Pacific Decadal Oscillation; and SOI, Southern Oscillation Index). The thick black contours depict the 5% significance level, highlighting the areas of strongest correlation. Here, WTC and XWT denote wavelet coherence and cross wavelet transform, respectively.

The cross-wavelet energy spectrum revealed a remarkable resonance period of 10.5 to 13.1 months (2001−2019) between NDVI and AMO in the MLYRB. The period between 2008 to 2012 notably exhibited a high-energy region, and the phase difference pointed towards a positive correlation between NDVI and AMO (**Figure 9A**). Moreover, there were two distinct resonance periods identified between NDVI and NAO: 9.0 to 12.5 months (2001−2009) and 9.2 to 12.7 months (2010−2019). Predominantly, the high-energy region was evident from 2011 to 2017, and the phase difference indicated a negative correlation between NDVI and NAO (**Figure 9B**). Furthermore, there were two significant resonance periods uncovered between NDVI and PDO: 8.8 to 14.7 months (2001−2008) and 8.6 to 12.9 months (2009−2019). The year 2005 primarily witnessed a high-energy region, and the phase difference implied that NDVI responded with a delay to alterations in PDO (**Figure 9C**). Lastly, two significant resonance periods were identified between NDVI and SOI: 11.8 to 13.0 months (2001−2003) and 10.3 to 11.8 months (2005−2016). The former high-energy region was primarily observed from 2001 to 2002, while the latter was focused in 2007. The phase difference hinted at a negative correlation between NDVI and SOI (**Figure 9D**).

In the low-energy region of the wavelet coherence power spectrum, NDVI exhibited a significant resonance period with AMO, spanning from 10.3 to 13.5 months (2008−2012). Notably, the phase difference was predominantly positive (**Figure 9E**), suggesting that a positive influence of AMO on the periodic variation of NDVI. Similarly, NDVI demonstrated a pronounced resonance period with NAO between 8.3 and 13.0 months (2014−2017). The phase difference was significantly negative (**Figure 9F**), implying that NDVI was strongly impacted by the counteracting effects of the NAO. Notably, a significant resonance period was observed between NDVI and PDO for a duration of 10.4 to 15.7 months (2005). This resonance was characterized by an upward phase difference, indicating that NDVI responded with a lag to PDO changes (**Figure 9G**). Additionally, NDVI displayed a significant resonance period with SOI from 3.3 to 4.7 months (2018−2019) (**Figure 9H**). The phase difference was markedly positive, showing that SOI facilitated the enhancement of NDVI.

#### 4.4.3 Impact of urbanization on NDVI

**Figure 10:**
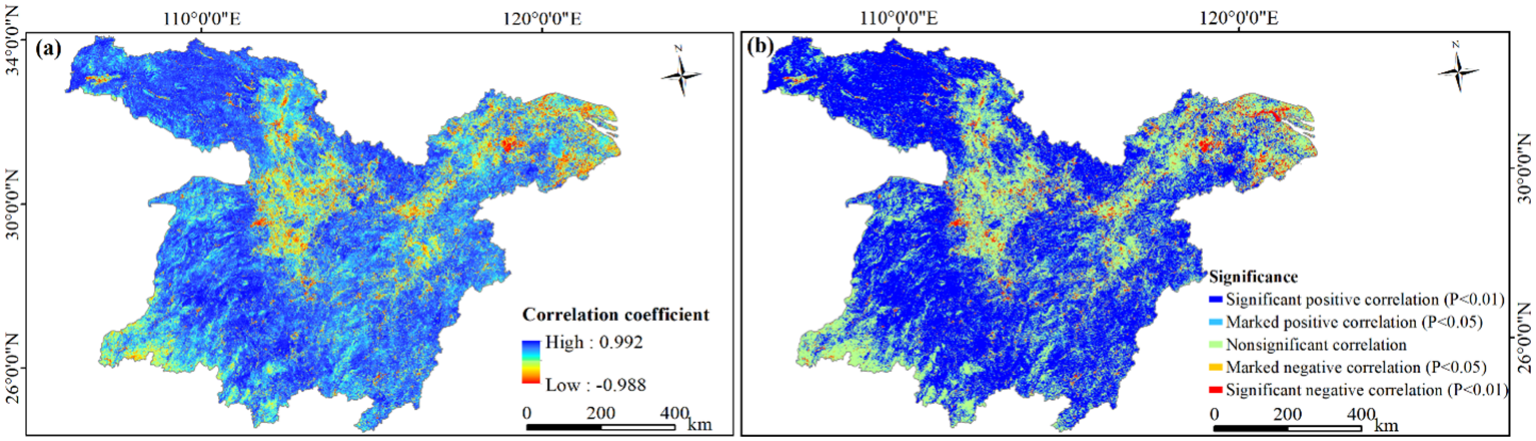
Correlation between NDVI and urbanization rate in the study area, 2003−2020. **(A)** Correlation coefficient; **(B)** significance of correlation.

The correlation between urbanization and NDVI exhibited notable spatial heterogeneity. Among the examined areas, 86.43% demonstrated a positive correlation between NDVI and urbanization rate, whereas 13.57% showed a negative correlation (**Figure 10A**). Specifically, 65.18% of these areas displayed a marked or significant positive correlation (**Figure 10B**). Notably, 54.32% presented a significant positive correlation, predominantly distributed in regions like Qinling-Daba Mountains, Wuling-Xuefeng Mountains, Dabie Mountains, Jiangnan Hills, Southern Anhui Mountainous Areas, and Western Zhejiang Hills. The remaining 10.86% had a marked positive correlation scattered across the study area. These mountainous regions had relatively lower urbanization rates. Simultaneously, the Chinese government’s emphasis on ecological environment protection has led to the implementation of various ecological preservation such as natural forest conservation and converting farmland back to forests, positively impacting NDVI.

Areas exhibiting a significant or marked negative correlation accounted for 4.30% (**Figure 10B**). Specifically, 2.73% showed a significant negative correlation, mainly distributed in the WMA, the CZTUA, the PLUA, and the YRD urban agglomeration. The 1.57% areas with a marked negative correlation were primarily concentrated in Lianghu Plain and along the Yangtze River. With urban expansion and rapid population growth, these regions saw rising urbanization rates, leading to the conversion of natural vegetation into urban land. This transformation resulted in reduced vegetation cover. Consequently, NDVI is negatively correlated with the urbanization rate.

**Figure 11:**
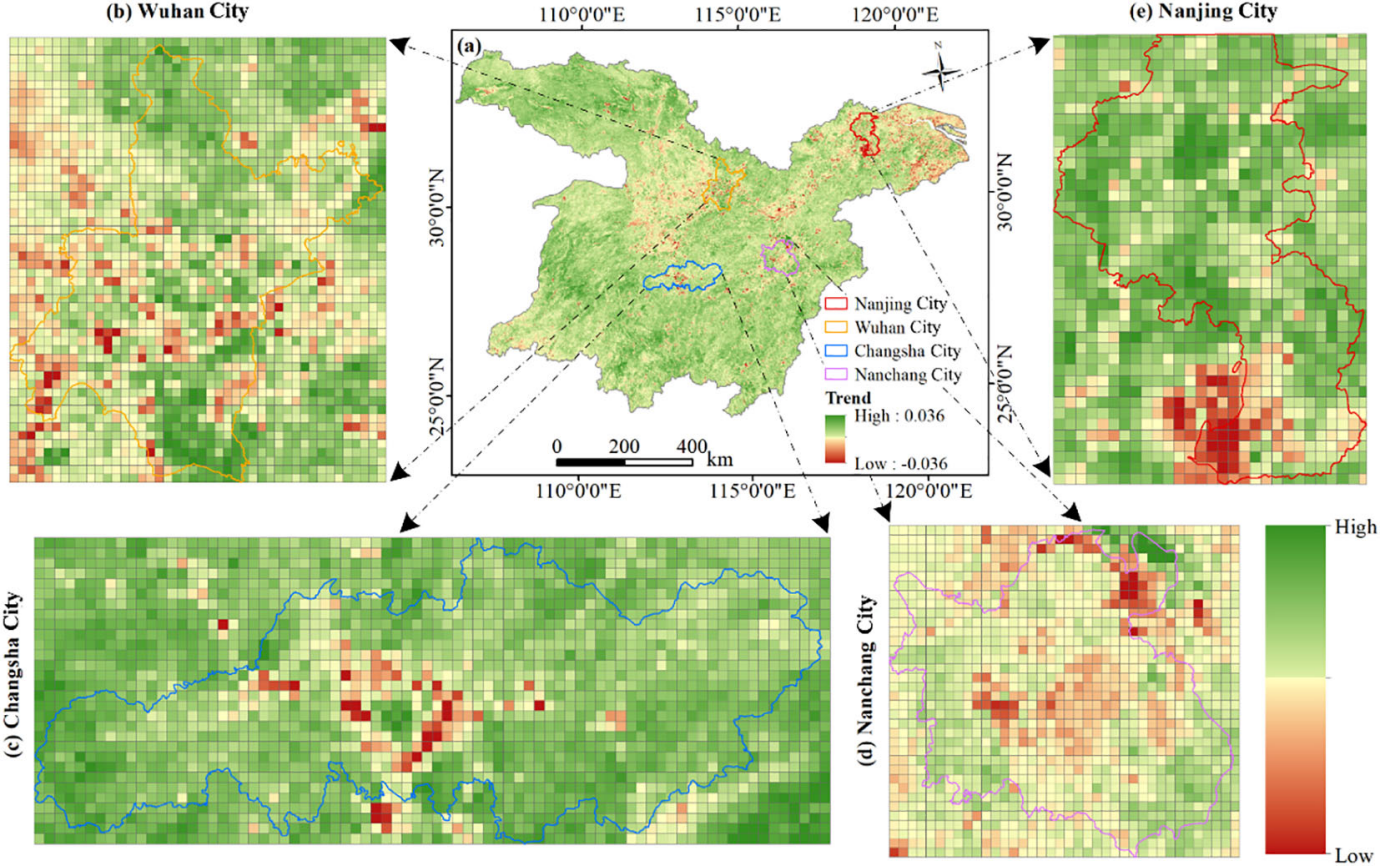
Impact of urban expansion on NDVI change rate. **(A)** Variations in NDVI, as well as exemplary cities such as **(B)** Wuhan; **(C)** Changsha; **(D)** Nanchang; and **(E)** Nanjing.

This study focused on the provincial capital cities of Wuhan (**Figure 11B**), Changsha (**Figure 11C**), Nanchang (**Figure 11D**), and Nanjing (**Figure 11E**) within the study area (**Figure 11A**) to examine effects of urban expansion on vegetation. By monitoring the NDVI change rate, it was observed that the NDVI generally exhibited a negative trend in the outskirts of each provincial capital. Specifically, the western region of Wuhan, the central area of Changsha, and the southern part of Nanjing all displayed a decreasing NDVI. Similarly, most areas in Nanchang also demonstrated a decline in NDVI, suggesting significant vegetation degradation in these regions. With the accelerating pace of urbanization, urban development continued to encroach upon the periphery of these cities. This haphazard expansion often came at the expense of the surrounding vegetation. Consequently, this destruction led to direct changed in land cover, resulting in a decrease in NDVI. This observation underscores the contribution of urban expansion to vegetation degradation.

### 4.5 Future trends of NDVI

**Figure 12:**
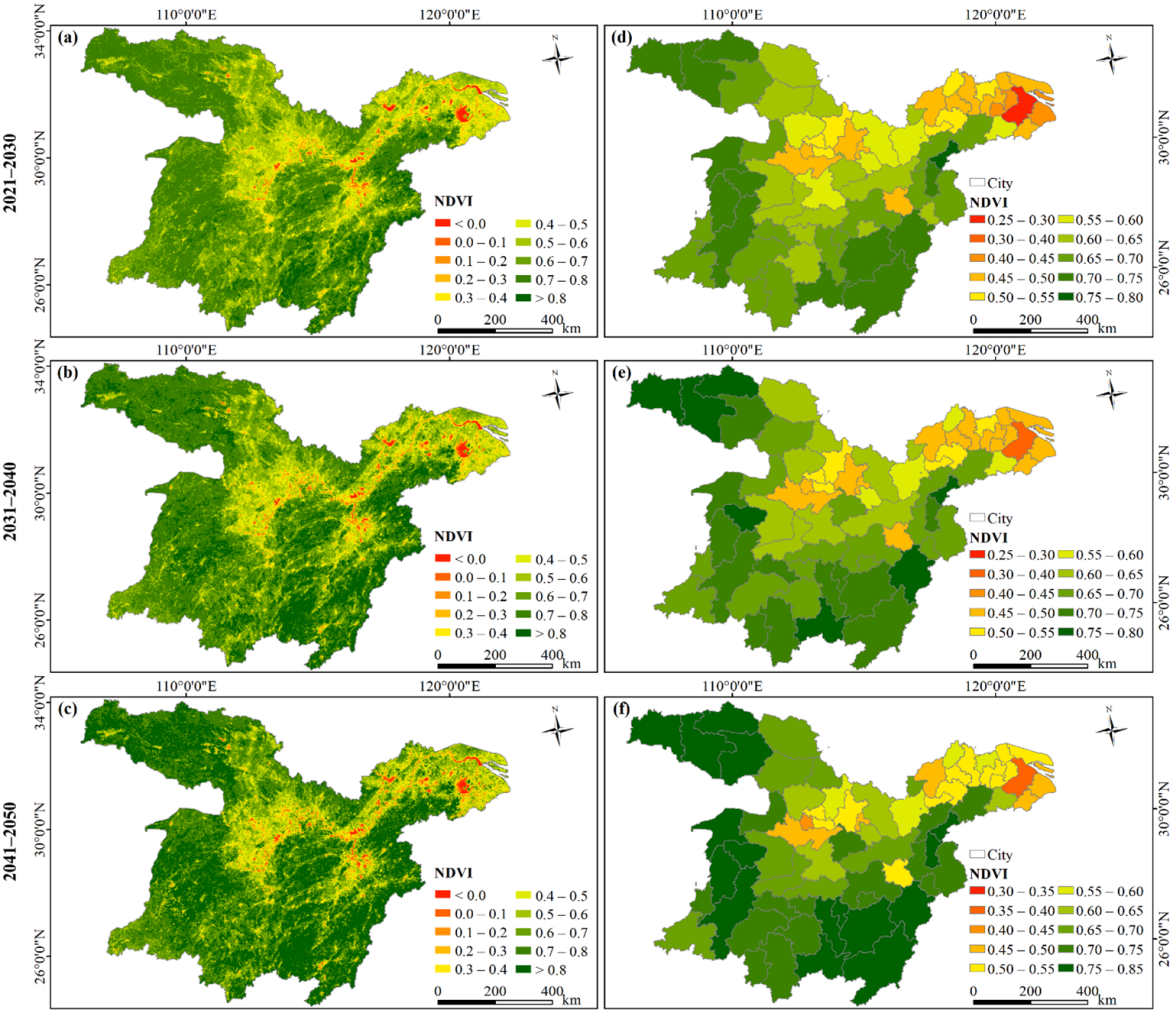
Spatial patterns of future NDVI based on **(A−C)** the whole-domain scale and **(D−F)** the city scale for the periods of 2021−2030, 2031−2040, and 2041−2050, respectively.

The proportions of regions with high NDVI values (> 0.6) within the study area are 67.35%, 72.72%, and 76.02% for the periods of 2021−2030 (**Figure 12A**), 2031−2040 (**Figure 12B**), and 2041−2050 (**Figure 12C**), respectively. These proportions suggest that the vegetation coverage in the study area is projected to continue on a greening trend, especially in the western region and Jiangnan Hills. This trend holds great significance not only for maintaining regional ecological balance and regulating climate but also offering robust support towards achieving China’s dual carbon goals. Areas with lower vegetation coverage are mainly located in the urban agglomeration in the middle reaches of Yangtze River, the Chaohu Lake Basin, the Taihu Lake Basin, and the YRD. These locations are characterized by rapid economic progress, a sizable population, and a relatively advanced level of urbanization, resulting in a degradation in vegetation (Qu et al., 2020).

**Figures 12D–F** illustrate a steady growth trend in NDVI across all cities spanning from 2021 to 2050. The proportions of cities where the NDVI exceeds 0.6 stand at 58.82%, 63.24%, and 66.18% for the decades 2021−2030, 2031−2040 and 2041−2050, respectively. Throughout the study period referenced, the NDVI of Huangshi consistently tops the list with the highest NDVI, followed by Ganzhou, Chenzhou, and Shangluo. In contrast, Suzhou persistently registers the lowest NDVI. Other highly urbanized areas, such as Shanghai Municipality, Wuxi, and Jiaxing, also exhibit relatively low NDVI values.

## 5 Discussion

### 5.1 NDVI variability

This study explores the spatiotemporal variations of vegetation in the MLYRB using multi-temporal remote sensing data. The results indicate a significant improvement in vegetation over the past 21 years, corroborating the conclusions of previous studies (Yi et al., 2021b; Zhang et al., 2023). Specifically, there has been a marked enhancement in vegetation coverage, particularly in the western and central regions of the study area. As suggested by some researchers, this pronounced trend can be attributed to the conversion of a significant portion of croplands into forests (Zhu et al., 2016). Also, climatic factors and ecological conservation efforts have positively contributed to this vegetation growth (Zhang et al., 2020). The NDVI has shown significant improvement in areas constituting 62.65% of the study area, especially in the DLB, PLB, and HRB. Since these three basins are pivotal commodity grain production centers in China, the observed NDVI dynamics align with changes in crop patterns (Han et al., 2020; Liu et al., 2021; Long et al., 2021). However, vegetation degradation accounted for merely 3.57% of the study area, primarily affecting urban areas. This result underscores the profound negative impact of urban expansion on vegetation coverage (Guan et al., 2019).

In this study, the SARIMA model was utilized to project future NDVI trends over multiple periods. The model relies on multiple non-seasonal and seasonal parameters. Notably, their estimation is solely based on a historical dataset, which may be influenced by factors such as sample size, data quality, and data distribution characteristics. Consequently, these estimations inherently carry a certain degree of uncertainty, thereby affecting the predictive accuracy. Nonetheless, the SARIMA model has been verified to exhibit high stability, and its predictive approach integrates both numerical values and noise, resulting in simulated values remarkably close to the actual ones (Jiang et al., 2010). This conclusion indicates that parameter estimation has a negligible effect on the model’s predictive accuracy.

### 5.2 Driving mechanisms of vegetation dynamics

A thorough comprehension of the relationship between climatic factors and NDVI is crucial for predicting vegetation dynamics and managing ecological restoration projects. Among various climatic factors, temperature and precipitation play a vital role in shaping regional NDVI variations (Li et al., 2021a, 2021). Numerous studies have predominantly focused on exploring the effects of temperature and precipitation on NDVI by calculating correlation coefficients (Gao et al., 2022; Yu et al., 2020). This research reveals a predominantly positive correlation between NDVI and temperature, echoing previous research findings (Hua et al., 2017; Zhang et al., 2020). Furthermore, moderate wind speeds expedite the transpiration rate of plant leaves, elevate CO_2_ levels within plant cells, enhance the net photosynthetic rate, and subsequently foster vegetation growth (Zhang et al., 2022b). Conversely, excessive precipitation can lead to vegetation root rot, while rainy conditions diminish solar radiation reaching the earth’s surface, ultimately impeding vegetation growth (Hussien et al., 2023).

Urban expansion profoundly impacts vegetation dynamics (Du et al., 2019a). As depicted in **Figure 12**, the vegetation in the primary provincial capitals and adjacent regions of the MLYRB exhibits a marked degradation trend. This degradation is largely attributed to the adverse effects of urban expansion. The negative correlation between urban development and vegetation can be ascribed to factors such as environmental deterioration, habitat loss, and changes in land cover during the urbanization process (He et al., 2014; Kowarik, 2011). Furthermore, vegetation changes are associated with different stages of urbanization (Fu et al., 2018). Cities in their early development stage often reduce vegetation cover to meet residential space demands (Wu et al., 2019). Conversely, more mature cities may prioritize increasing urban green spaces, which could facilitate vegetation regeneration. Hence, as the demand for better urban living standards persists, vegetation degradation will gradually give way to improvement (Li et al., 2020).

Climatic variables, including temperature, precipitation, wind speed, relative humidity, and sunshine duration, directly shape the growth cycle, water demand, and light conditions of vegetation (Wang et al., 2024). Meanwhile, human activities primarily impact vegetation changes through land use/cover changes (Yin et al., 2018), population migrations (Inderjit et al., 2017), urban expansion (De La Barrera and Henríquez, 2017), and artificial afforestation (Fu et al., 2024). These anthropogenic effects are often localized and direct, intricately tied to the intensity and patterns of human intervention. In summary, vegetation change is a multifaceted phenomenon influenced by various factors, and the degree of this influence varies across regions. Notably, climatic factors exert a long-term influence on vegetation changes, whereas human activities typically have a more immediate and visible impact. Consequently, it is imperative to separately explore the effects of climate factors and human activities on vegetation changes to gain a deeper understanding of their respective mechanisms and impact levels.

### 5.3 Limitations and future prospects

Examining the regional patterns of vegetation dynamics and their responses to climate change aids in gaining a better understanding of the driving forces behind vegetation variability. Nevertheless, in exploring the impacts of climate change on vegetation, this study neglected the time lag and cumulative effects of climatic factors on vegetation growth. Obviously, the significance of these time lags and cumulative effects has been verified in numerous studies (Wu et al., 2015; Zuo et al., 2021). Meanwhile, this research has not fully considered other potential factors influencing vegetation dynamics, such as CO_2_ fertilization (Zhu et al., 2016), extreme climatic events (Zscheischler et al., 2014), and anthropogenic activities (Jiang et al., 2017). Additionally, the period for the urbanization rate used in this study spans from 2003 to 2020, with missing statistical information for the preceding three years (2000−2002), might undermine the reliability of evaluating the impact of urbanization on vegetation changes. Consequently, future research could endeavor to develop a comprehensive model capable of quantifying most relevant factors to explore the driving mechanisms of vegetation dynamics at regional and even global scales.

## 6 Conclusion

This study investigated the impact of climate change and urbanization on NDVI variations in the MLYRB, and projected future NDVI trends. The primary findings are summarized below:

1. The annual average NDVI in the study area exhibited a significant upward trend from 2000 to 2020. This growing trend was evident in 89.38% of the area, with western and southern cities boasting significantly higher NDVI values than their counterparts. Meanwhile, 67.76% of the area underwent significant NDVI changes, with extremely significant increases and decreases accounting for 53.56% and 2.59%, respectively. Over the study period, the centroid of NDVI consistently displaced, with the migration distance progressively increasing, underscoring the significant NDVI variations.
2. Temperature and wind speed positively impacted 32.54% and 20.92% of the study area, respecti vely. Conversely, 11.63% of the area was adversely affected by sunshine duration. Precipitation and relative humidity exerted a comparatively minor effect on vegetation. Global climate oscillations are capable of influencing periodic variations in NDVI. Additionally, despite urbanization being a pivotal contributor to NDVI decline, a positive correlation between the two was noted.
3. Vegetation is anticipated to sustain its greening trend in the foreseeable future, evident in the increasing NDVI values across all cities. Nevertheless, areas with low vegetation cover remain predominantly in highly urbanized zones.

## Data Availability

The original contributions presented in the study are included in the article/supplementary material. Further inquiries can be directed to the corresponding author.
